# Low-Symmetry Mixed Fluorinated Subphthalocyanines as Fluorescence Imaging Probes in MDA-MB-231 Breast Tumor Cells

**DOI:** 10.3390/ijms18061177

**Published:** 2017-06-01

**Authors:** Katherine J. McAuliffe, Megan A. Kaster, Regina G. Szlag, Evan R. Trivedi

**Affiliations:** Department of Chemistry, Oakland University, Rochester, MI 48309, USA; kjmcauliffe@oakland.edu (K.J.M.); makaster@oakland.edu (M.A.K.); rgszlag@oakland.edu (R.G.S.)

**Keywords:** subphthalocyanine, fluorescence imaging, ^19^F NMR, fluorescence microscopy

## Abstract

Boron subphthalocyanines (SPcs) are aromatic macrocycles that possess a combination of physical and optical properties that make them excellent candidates for application as fluorescent imaging probes. These molecules have intense electronic absorption and emission, and structural versatility that allows for specific tuning of physical properties. Herein, we report the synthesis of a series of low-symmetry fluorinated SPcs and compare them to analogous compounds with varying numbers of peripheral fluorine atoms and varied aromaticity. Across the series, with increasing addition of fluorine atoms to the periphery of the ring, a downfield chemical shift in ^19^F NMR and a bathochromic shift of electronic absorption were observed. Expanding the size of the aromatic ring by replacing peripheral benzo- groups with naphtho- groups prompted a far more drastic bathochromic shift to absorption and emission. Fluorescence quantum yields (Φ*_f_*) proved to be sufficiently high to observe intracellular fluorescence from MDA-MB-231 breast tumor cells in vitro by epifluorescence microscopy; fluorination proved vital for this purpose to improve solubility. This report lays the groundwork for the future development of these promising SPcs for their ultimate application as near-infrared (NIR) fluorescent imaging probes in biological systems.

## 1. Introduction

Boron subphthalocyanines (SPcs) are 14π-electron tripyrrolic macrocycles that were first discovered in 1972 [1], and have garnered significant attention for their structural versatility and optical properties, which has recently been reviewed extensively [2,3]. The ring system is conical in shape with an apical central boron with an axial ligand, usually a halogen (*X* = F, Cl, Br) [4], making these macrocycles more polar and less aggregative than their planar tetrapyrrole phthalocyanine (Pc) cousins. Like Pcs, SPcs are synthesized by the templated cyclization of phthalonitrile derivatives. Synthesis from a C_2v_ symmetric phthalonitrile yields C_3v_ symmetric SPcs, whereas lower symmetry phthalonitrile starting materials yield a mixture of constitutional isomers (C_1_ and C_3_) that are axially chiral [5]. Low-symmetry elements can also be introduced through a mixed cyclization with multiple phthalonitrile derivatives; co-cyclization of phthalonitrile with naphthalene-2,3-dicarbonitrile, for example, yields a series of four macrocycles denoted A_3−*n*_B*_n_*(SPc) where “A” is a peripheral benzo moiety and “B” is a peripheral naphthalene [6]. Similar strategies have been employed to produce fluorinated A_2_B and AB_2_ type SPcs [7], designer pyrene fused SPcs [8], and even core-expanded analogues with annulated 6- and 7-membered rings [9]. We are particularly interested in using a cross-cyclization approach to synthetically tune SPc properties for application as fluorescent tumor cell imaging probes.

Fluorescence imaging with a contrast agent is a growing technique for the study and diagnosis of cancer [10,11,12]. A fluorescence probe that absorbs and emits in the near-infrared (NIR) is particularly valuable due to the ability for NIR light to effectively penetrate soft tissue [13]. One promising application of this technology is in the real-time intraoperative detection of cancer in humans [14,15], a prospect that would greatly aid surgical oncologists. Development of new contrast agents with optimal photophysical properties and some sort of preferential tumor accumulation is, therefore, imperative.

Pyrrolic macrocycles have intense absorption and fluorescence [16], warranting their application as biomedical fluorescence imaging agents. Selected Pc derivatives have seen success on this front [17,18,19,20]; far fewer examples of SPc fluorescence within cells exist in the literature [21,22]. In each SPc case, the axial ligand of boron is exchanged to improve solubility. Herein, we describe a series of mixed SPcs and slightly larger subnaphthalocyanines (SNcs) with a variable number of peripheral fluorine atoms (# *F* = 0, 4, 8, or 12). Fluorine was chosen here for its ability to bathochromically shift photophysical properties toward the NIR [23,24], the pharmacological inertness of the C–F bond in drug design [25], and the effects that a variable number of fluorines will have on lipophilicity and biodistribution within the cell [26]. Relevant photophysical properties have been tabulated and preliminary studies of intracellular fluorescence is reported.

## 2. Results and Discussion

### 2.1. Synthesis of SPcs

**Figure 1:**
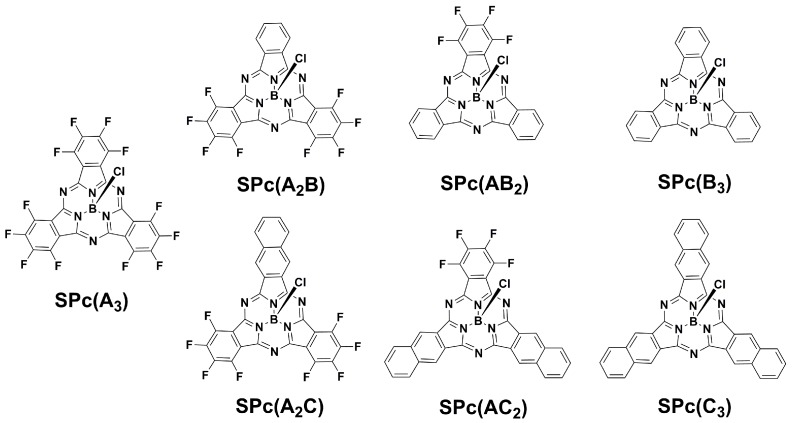
Mixed fluorinated boron subphthalocyanines (SPcs).

Two types of macrocycles have been synthesized with variable peripheral groups of the form SPc(A*_n_*B_3−*n*_) and SPc(A*_n_*C_3−*n*_), where “A” is tetrafluorobenzo-, “B” is benzo-, “C” is naphtho-, and *n* is an integer 0–3 (Figure 1). A modified literature procedure was employed, whereby aromatic dinitriles were heated to 140 °C in *p*-xylene with an excess (1.5 equivalents) of BCl_3_. The previously reported compounds SPc(A_3_) [27], SPc(B_3_) [1], and SPc(C_3_) [6], can be prepared from the lone cyclization of tetrafluorophthalonitrile, phthalonitrile, and naphthalene-2,3-dicarbonitrile, respectively; mixed “AB” and “AC” SPcs were prepared by co-cyclizing dinitriles with varying stoichiometry. While the “AC” series of SPcs have been previously reported [7], SPc(A_2_B) and SPc(AB_2_) are novel so their synthesis will be discussed in more detail.

Co-cylization with equimolar amounts of tetrafluorophthalonitrile and phthalonitrile yields all four SPc(A*_n_*B_*n*−3_). In order to optimize yields, separate reactions were completed using a 3:1 stoichiometric ratio of one cyclization partner to the other. An excess of phthalonitrile yielded SPc(AB_2_) in *ca.* 10% yield, whereas an excess of tetrafluorophthalonitrile yielded SPc(A_2_B) at *ca.* 30%. We attribute the disparity in yield to the relative cyclization rates of the two starting materials. In sub-stoichiometric co-cyclization reactions, SPc(A_3_) was observed by TLC but not collected. Purification by flash chromatography of the SPc mixtures was achieved and identity and purity were confirmed by HRMS, NMR (^19^F, ^1^H), and analytical HPLC (Figures S1–S8).

### 2.2. ^19^F NMR Characterization of SPcs

One convenient benefit of fluorinated SPcs with varying symmetries is that each would be expected to have a signature ^19^F NMR profile. Each SPc contains two types of fluorine atoms, α-F (non-peripheral) and β-F (peripheral), relative to the central pyrroles. Based on ^19^F NMR trends observed for various substituted fluorobenzenes [28], and analogous ^1^H NMR data for SPcs [2], we assign the non-peripheral F-atoms to be further upfield (more negative ppm) than peripheral F-atoms (Table 1). As expected, the C_3v_ SPc(A_3_) and two C_s_ SPc(AX_2_) exhibited two signals that appeared as doublets with J_FF_~17 Hz. The expected meta-coupling was not resolved. The two C_s_ SPc(A_2_X) compounds exhibited four signals, two in the α-F region and two in the β-F region, as complex multiplets. The general trend across the series is that the fluorine atoms provide a deshielding effect, with the signal from the most fluorinated SPc appearing the furthest downfield (δ −136.7). The naphtho- functionalized “AC” SPcs are more shielded than the analogous benzo- “AB” SPcs with the furthest upfield signal being for SPc(AC_2_). These data not only confirm identity of the fluorinated SPcs studied here, but also provide a library of ^19^F chemical shifts for SPcs to be used in future synthetic refinement.

### 2.3. Photophysical Properties of SPcs

**Figure 2:**
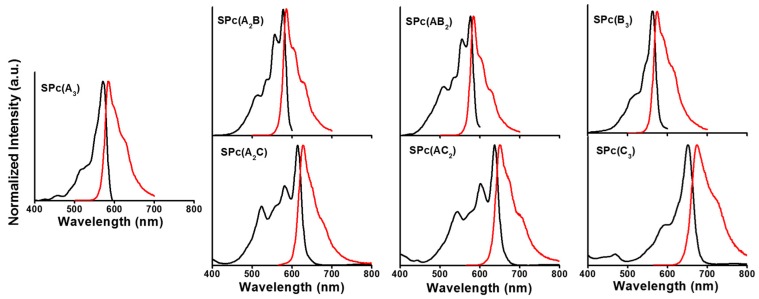
Electronic absorption (**black**) and emission (**red**) spectra for mixed fluorinated SPcs.

Much like the more common porphyrins and Pcs, SPcs possess two major absorptions [29]: the Soret-band in the UV region (~300–400 nm) and the Q-band in the visible (>500 nm). The Q-band is sensitive to both peripheral functionalization and symmetry of the macrocycle. Peripheral functionalization has potential to bathochromically shift absorption toward the NIR and symmetry has effects on the shape of the spectrum; low-symmetry macrocycles show splitting of the Q-band due to lower degeneracy of the lowest unoccupied molecular orbital (LUMO). Indeed, the perfectly symmetrical (C_3v_) SPcs (SPc(A_3_), SPc(B_3_), and SPc(C_3_)) exhibited a single peak in the Q-band region, whereas low-symmetry (C_s_) SPcs (SPc(A_2_B), SPc(AB_2_), SPc(A_2_C), and SPc(AC_2_)) showed more complex splitting of the Q-band (Figure 2). Fluorescence (λ*_f_*) occurs slightly red-shifted from the Q-band absorption (λ_max_), the difference in energy between the two being termed the Stokes shift. The efficiency of fluorescence can be quantified by the quantum yield of fluorescence (Φ*_f_*). Each of these parameters are summarized in Table 2 in tetrahydrofuran (THF) solution.

For the “AB” series of SPcs, the shifting of peak positions was dominated by the addition of fluorine. The electron withdrawing groups stabilize the LUMO, thereby red-shifting the emission as far as ca. 585 nm. For the “AC” series, the effect of adding additional fused benzo rings was far greater than that of peripheral fluorine. Each successive addition of a fused benzo ring red-shifted the emission by over 20 nm, despite the deletion of electron withdrawing fluorine atoms. This side by side comparison of “AB” and “AC” SPcs demonstrates the synthetic tunability of optical properties, with two members of the series (SPc(AC_2_) and SPc(C_3_)) approaching the edge of the NIR window of maximum light penetration. Quantum yields were nearly consistent across the series, within the error of the measurement, with a few outliers on the low-end. Quantum yields will be discussed in more detail below with respect to in vitro cell imaging studies below.

### 2.4. In Vitro Epifluorescence Microscopy in MDA-MB-231 Breast Tumor Cells

While the majority of compounds discussed here are unsuitable for ultimate application in the NIR region, proof of concept experiments were conducted by epifluorescence microscopy to judge how synthetic modifications may influence cellular uptake and/or biodistribution. The “AC” series of compounds, while their optical properties are closer to ideal, proved insufficiently soluble in aqueous cell culture medium for testing. The “AB” series of SPcs made stable solutions in media with the addition of 0.2% dimethylsulfoxide (DMSO) as a solubilizing agent. Compounds such as these, at the edge of the usable solubility range, have greater potential to interact with hydrophobic proteins and membranes within the cell, which can positively effect biodistribution and uptake.

**Figure 3:**
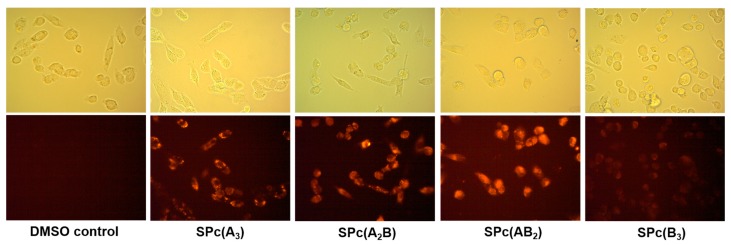
Brightfield (**upper**) and epifluorescence ((**lower**) λ_ex_ = BP 528–553 nm, λ_em_ = BP 578–633 nm) of MDA-MB-231 breast tumor cells treated with 50 μM SPc for 15 min (400×).

The MDA-MB-231 breast tumor cells were plated and allowed to grow to 50% confluency before being treated with SPc solutions (50 µM) for 15 min (Figure 3). Brightfield and epifluorescence micrographs were collected on the live cells using an orange TRITC filter set. Fluorinated SPcs all showed strong intracellular fluorescence relative to DMSO treated control cells, whereas the signal from SPc(B_3_) was far weaker, albeit present. Two factors dictate the brightness of cell images: (1) the extent of cellular uptake and (2) the quantum yield of the probe. Considering that the quantum yields of these SPcs are within the same order of magnitude, one can assume that the cellular uptake of SPc(B_3_) is far less than the others under the same conditions. This observation is not particularly surprising since the SPc(B_3_) is devoid of solubilizing fluorine atoms. The key observation here that will guide future synthetic refinement is that simple addition of fluorine atoms to the periphery of the SPc can drastically change biocompatibility.

## 3. Materials and Methods

### 3.1. Materials

Reagents and chemicals, including silica gel (60 Å, 230–400 mesh), were purchased from VWR International (Radnor, PA, USA) and used without further purification unless otherwise noted. Tetrafluorophthalonitrile (TFPN) was purchased from TCI America (Portland, OR, USA) and boron subphthalocyanine chloride (SPc(B_3_)) and boron subnaphthalocyanine chloride (SPc(C_3_)) were purchased from Sigma-Aldrich (St. Louis, MO, USA).

### 3.2. General Considerations

Nuclear magnetic resonance (NMR) spectra were recorded on an Avance III (400 MHz, Bruker) spectrophotometer. ^19^F NMR spectra were recorded using trifluoroacetic acid as a standard (δ = −76.55 ppm). Mass spectrometry was performed on an LC/MS 6545 Q-TOF (Agilent, Santa Clara, CA, USA) in APCI mode. Purity analysis by HPLC was performed on an Agilent 1100 system with diode array detector and C8 ZORBAX Eclipse Plus column (Agilent). Absorption data was collected on a Cary-100 UV–Vis spectrophotometer (Agilent) in double-beam mode using 1 cm path quartz cuvettes. Corrected fluorescence spectra were collected on a Fluorolog 3 fluorometer (Horiba Jobin-Yvon, Edison, NJ, USA) equipped with an R928 PMT (Hamamatsu, Shizuoka, Japan). Solutions were prepared such that absorption remained below 0.1 AU to prevent reabsorption and self-quenching. Fluorescence spectra were recording using an excitation wavelength corresponding to the maximum Q-band absorption.

### 3.3. Synthesis

Naphthalene-2,3-dicarbonitrile [30], boron 1,2,3,4,8,9,10,11,15,16,17,18-dodecafluorosubphthalocyanine chloride (SPc(A_3_)) [27], boron 1,2,3,4,8,9,10,11-octafluoro-naphtho[*b*] subphthalocyanine chloride (SPc(A_2_C)), boron 1,2,3,4-tetrafluoro-dinaphtho[*b*,*g*] subphthalocyanine chloride (SPc(AC_2_)) were prepared by literature procedure [7]. Hitherto unreported characterization data (^19^F NMR) are summarized for SPc(A_3_), SPc(A_2_C), and SPc(AC_2_).

***SPc(A_2_B)****—Boron 1,2,3,4,8,9,10,11-octafluorosubphthalocyanine chloride*. To a 25 mL roundbottom flask was added phthalonitrile (0.091 g, 0.71 mmol) and tetrafluorophthalonitrile (0.422 g, 2.11 mmol). The flask was purged with N_2_, *p*-xylene (8 mL) was added, and the flask was heated to 140 °C. A solution of BCl_3_ (1 M in *p*-xylene, 1.05 mL, 1.05 mmol) was added, resulting in a yellow solution. After approximately five minutes, the solution changed color to dark red. The reaction was stirred for 40 min, after which the solvent was reduced in vacuo. The resulting dark purple solid was dissolved in dichloromethane and filtered through a plug of silica gel. The resulting sample was purified by flash chromatography on silica gel (1:9 ethyl acetate: hexane). A dark purple solid was obtained (0.129 g, 32%). ^1^H NMR (400 MHz, CDCl_3_) δ (ppm): 8.06 (dd, *J* = 6 Hz, 3 Hz, 2H), 8.94 (dd, *J* = 6 Hz, 3 Hz, 2H); ^19^F NMR (376 MHz, CDCl_3_) δ (ppm): −138.5 (m, 2F), −138.8 (m, 2F), −149.2 (m, 2F), −149.9 (m, 2F); HR-LCMS APCI: calcd. for (C_24_H_5_BClF_8_N_6_) [M + H]^+^, 575.0230; found, 575.0220; UV-vis (THF): λ_max_ (nm) (log ε (M^−1^·cm^−1^)), 298 nm (4.6), 576 nm (4.7).

***SPc(AB_2_)****—Boron 1,2,3,4-tetrafluorosubphthalocyanine chloride*. The synthesis was completed using an analogous procedure with the following proportions of reagents: phthalonitrile (0.273 g, 2.13 mmol), tetrafluorophthalonitrile (0.141 g, 0.704 mmol), *p*-xylene (10 mL), BCl_3_ (1 M in *p*-xylene, 1.5 mL, 1.5 mmol). A dark purple solid was obtained (0.031 g, 9%). ^1^H NMR (400 MHz, CDCl_3_) δ (ppm): 8.00–8.04 (m, 4H), 8.90–8.94 (m, 4H); ^19^F NMR (376 MHz, CDCl_3_) δ (ppm): −140.3 (d, *J* = 16 Hz, 2F), −151.7 (d, *J* = 16 Hz, 2F); HR-LCMS APCI: calcd. for (C_24_H_9_BClF_4_N_6_) [M + H]^+^, 503.0606; found, 503.0606; UV-vis (THF): λ_max_ (nm) (log ε (M^−1^·cm^−1^)), 306 nm (4.5), 572 nm (4.7).

***SPc(A_3_)****—Boron 1,2,3,4,8,9,10,11,15,16,17,18-dodecafluorosubphthalocyanine chloride*. 6 g scale, 17% yield; ^19^F NMR (376 MHz, CDCl_3_) δ (ppm): −136.7 (d, *J* = 17 Hz, 8F), −147.4 (d, *J* = 17 Hz, 8F).

***SPc(A_2_C)****—Boron 1,2,3,4,8,9,10,11-octafluoro-naphtho[b] subphthalocyanine chloride*. 2 g scale, 2% yield; ^19^F NMR (376 MHz, CDCl_3_) δ (ppm): −138.8 (m, 2F), −138.9 (m, 2F), −149.2 (m, 2F), −150.6 (m, 2F)

***SPc(AC_2_)****—Boron 1,2,3,4-tetrafluoro-dinaphtho[b,g] subphthalocyanine chloride*. 2 g scale, 3% yield; ^19^F NMR (376 MHz, CDCl_3_) δ (ppm): −141.8 (d, 17 Hz, 2F), −153.7 (d, 17 Hz, 2F)

### 3.4. Fluorescence Quantum Yield Determination

Fluorescence quantum yields were determined by the relative method [31], using cresyl violet (Φ*_f_* = 0.54, methanol) as a standard [32], and analysis with Equation (1) where *r* and *x* denote the standard and unknown, respectively, *A* is the absorption intensity at the excitation wavelength, *F* is the integrated fluorescence intensity, and *n* is the refractive index of the solvent. Cross calibration determined less than 10% error for this method and instrumentation.

(1) $$\Phi_{x}=\;\Phi_{r}\cdot \left(\frac{A_{r}\cdot F_{x}\cdot {n_{x}}^{2}}{A_{x}\cdot F_{r}\cdot {n_{r}}^{2}}\right)$$

### 3.5. Cell Culture and Fluorescence Imaging

Cell culture reagents were obtained from Hyclone (GE) unless otherwise noted. MDA-MB-231 breast tumor cells were obtained from American Type Culture Collection and maintained in Dulbecco’s Modified Eagle Medium (DMEM) supplemented with 10% fetal bovine serum. For imaging assays, cells were plated in 12-well plates (Corning, Corning, NY, USA) and allowed to grow to ca. 50% confluency before being treated with solutions of SPcs in media (0.2% dimethylsulfoxide). Images were collected with a VWR Inverted Fluorescence Microscope, Moticam 5.0 (Motic, Hong Kong) and TRITC filter set (λ_ex_ = 540 nm ± 12 nm, λ_em_ = 605 nm ± 27 nm).

## 4. Conclusions

We have reported here the synthesis of novel mixed fluorinated SPcs and provided a comparison to a number of known analogs of similar structure. Of particular note is the tabulation of the effects of step-wise synthetic modification on physical properties relevant to the development of NIR fluorescent probes based on this SPc platform. Fluorinated SPcs have been shown to be taken up by MDA-MB-231 breast tumor cells where significant intracellular fluorescence is observed. Future directions include further study into synthetic methods to bathochromically shift emission and improve solubility for ultimate application of this family of molecules as NIR fluorescent imaging probes.

## Figures and Tables

**Table 1:** ^19^F NMR data for fluorinated SPcs ^1^.

Compound	α-F–δ (ppm)	β-F–δ (ppm)
SPc(A_3_)	−147.4 (d) ^2^	−136.7 (d)
SPc(A_2_B)	−149.2 (m), −149.9 (m)	−138.5 (m), −138.8 (m)
SPc(AB_2_)	−151.7 (d)	−140.3 (d)
SPc(A_2_C)	−149.2 (m), −150.6 (m)	−138.8 (m), −138.9 (m)
SPc(AC_2_)	−153.7 (d)	−141.8 (d)

^1^ Samples in CDCl_3_ at 376.5 MHz. ^2^ multiplicity.

**Table 2:** Summary of SPc photophysical parameters ^1^.

Compound	Absorption (λ_max_, nm)	Emission (λ_f_, nm)	Stokes Shift (cm^−1^)	Quantum Yield (Φ*_f_*)
SPc(A_3_)	571	584	390	0.30
SPc(A_2_B)	578	585	210	0.19
SPc(AB_2_)	572	578	180	0.26
SPc(B_3_)	563	574	460	0.29
SPc(A_2_C)	613	628	390	0.21
SPc(AC_2_)	637	650	310	0.16
SPc(C_3_)	651	674	520	0.28

^1^ Samples in THF.

## Supplementary Materials

Supplementary materials can be found at www.mdpi.com/1422-0067/18/6/1177/s1.

## Abbreviations

SPc	Subphthalocyanine
Pc	Phthalocyanine
NIR	Near-infrared
LUMO	Lowest unoccupied molecular orbital
THF	Tetrahydrofuran
HPLC	High performance liquid chromatography
DMSO	Dimethylsulfoxide
DMEM	Dulbecco’s modified eagle medium
