# Supplementary material for: Low-Symmetry Mixed Fluorinated Subphthalocyanines as Fluorescence Imaging Probes in MDA-MB-231 Breast Tumor Cells
Source: Int J Mol Sci. 2017 Jun 1;18(6):1177. doi: 10.3390/ijms18061177 (PMC5486000; doi:10.3390/ijms18061177)
Supplement: Supplementary file 1 [file ijms-18-01177-s001.pdf]

Supplementary material for:

Low-symmetry mixed fluorinated subphthalocyanines as fluorescence imaging probes in MDA-MB-231 breast tumor cells.

Katherine J. McAuliffe,[1] Megan A. Kaster,[1] Regina G. Szlag,[1] and Evan R. Trivedi.[1*]

[1]Oakland University, Department of Chemistry, Rochester, MI 48309, USA

[*]Corresponding author: trivedi@oakland.edu

```
Purity results peak 1 at 3.434 min.

 Signal DAD1 A, Sig=254,4 Ref=off (RW\RW 2017-05-09 11-40-17\071-0101.D)
```

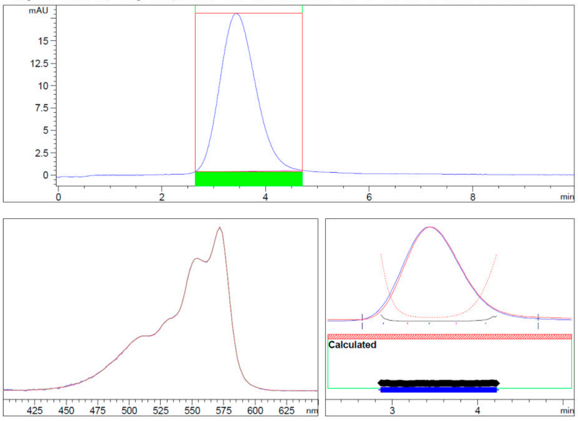


```
-> The purity factor is within the calculated threshold limit. <-

Purity factor : 999.986 (204 of 204 spectra are within the calculated threshold
                limit.)
Threshold     : 999.384  (Calculated with 204 of 204 spectra)
Reference     : Peak start and end spectra (integrated) (2.646 / 4.706)
Spectra       : 5  (Selection automatic, 5)
Noise Threshol: 0.100  (Set by user)

                          *** End of Report ***
```

Figure S1 – Purity report for **SPc(AB$_2$)**.

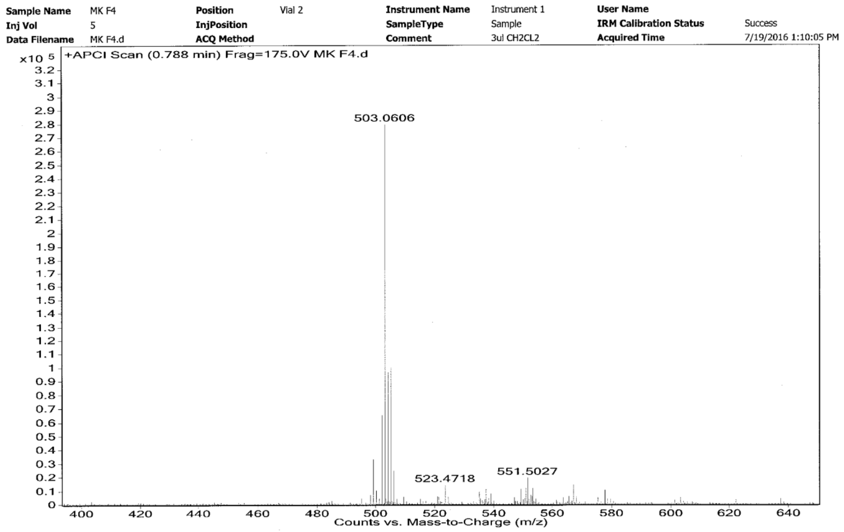

Figure S2 – HR-APCI-MS spectrum for **SPc(AB$_2$)**.

```
Purity results peak 1 at 3.099 min.

 Signal DAD1 A, Sig=254,4 Ref=off (RW\RW 2017-05-09 10-57-18\072-0201.D)
```

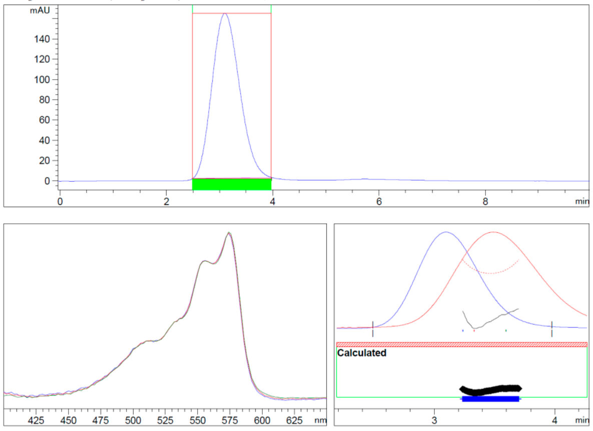


```
-> The purity factor is within the calculated threshold limit. <-

Purity factor : 999.739 (71 of 71 spectra are within the calculated threshold limit.)
Threshold     : 996.054  (Calculated with 71 of 71 spectra)
Reference     : Peak start and end spectra (integrated) (2.488 / 3.975)
Spectra       : 3  (Selection automatic, 5)
Noise Threshol: 0.100  (Set by user)

                        *** End of Report ***
```

Figure S3 – Purity report for **SPc(A$_2$B)**

**Sample Name**	MK F8	**Position**	Vial 3	**Instrument Name**	Instrument 1	**User Name**	
**Inj Vol**	5	**InjPosition**		**SampleType**	Sample	**IRM Calibration Status**	Success
**Data Filename**	MK F8.d	**ACQ Method**		**Comment**	3ul CH2CL2	**Acquired Time**	7/19/2016 1:13:12 PM

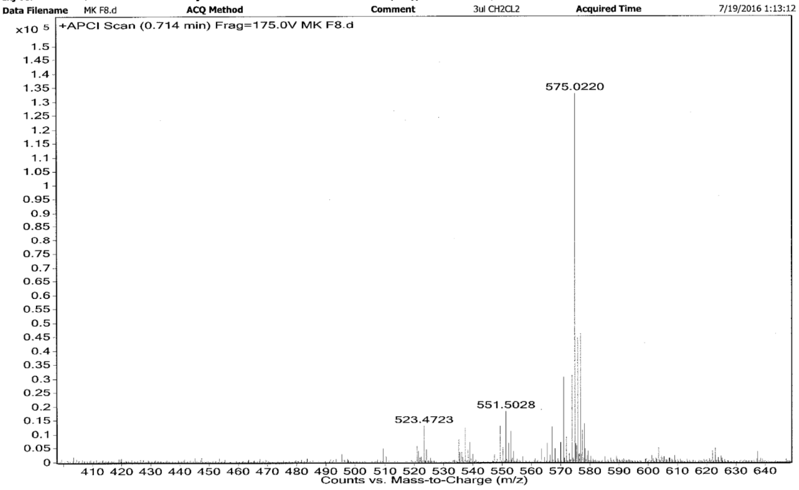

Figure S4 – HR-APCI-MS spectrum for **SPc(A$_2$B)**.

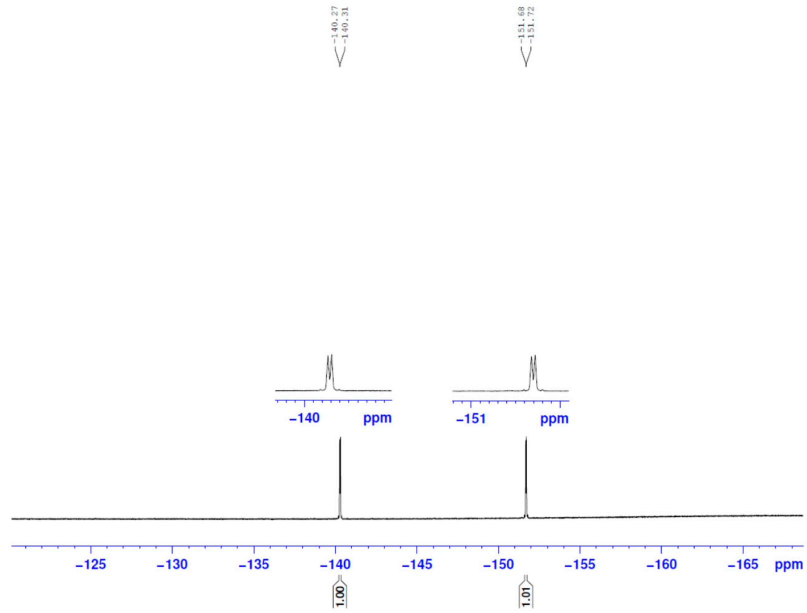

Figure S5 – ^{19}F NMR spectrum for SPc(AB$_2$)

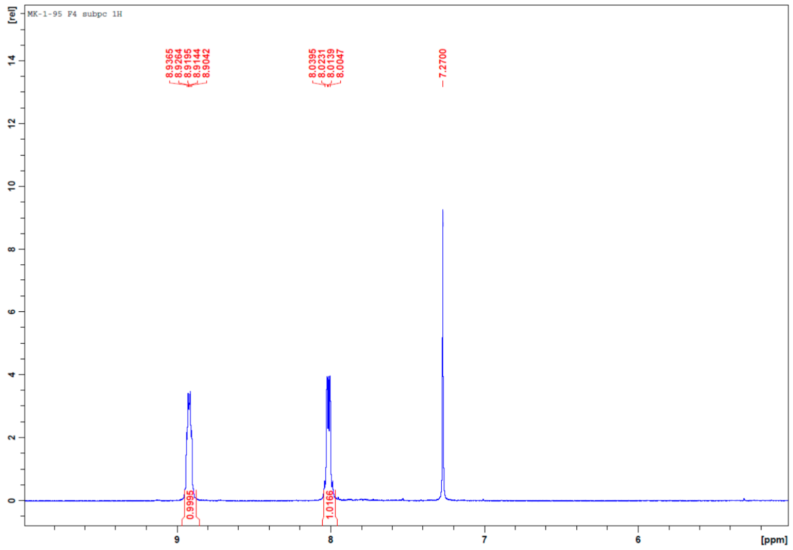

Figure S6 – ^{1}H NMR spectrum for SPc(AB$_2$) showing two sets of overlapping multiplets.

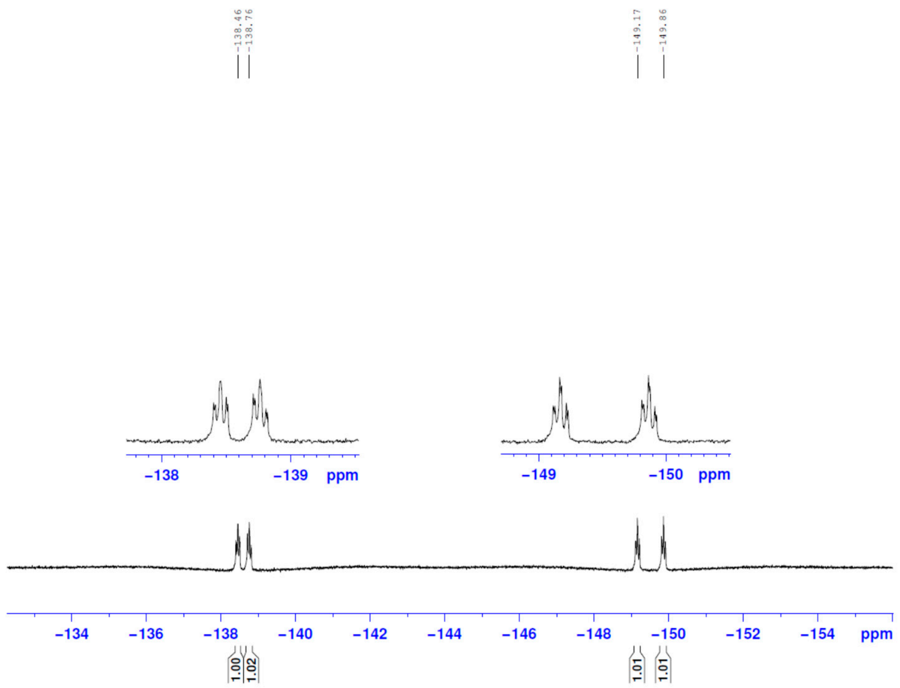

Figure S7 – ^{19}F NMR spectrum for SPc(A_2B)

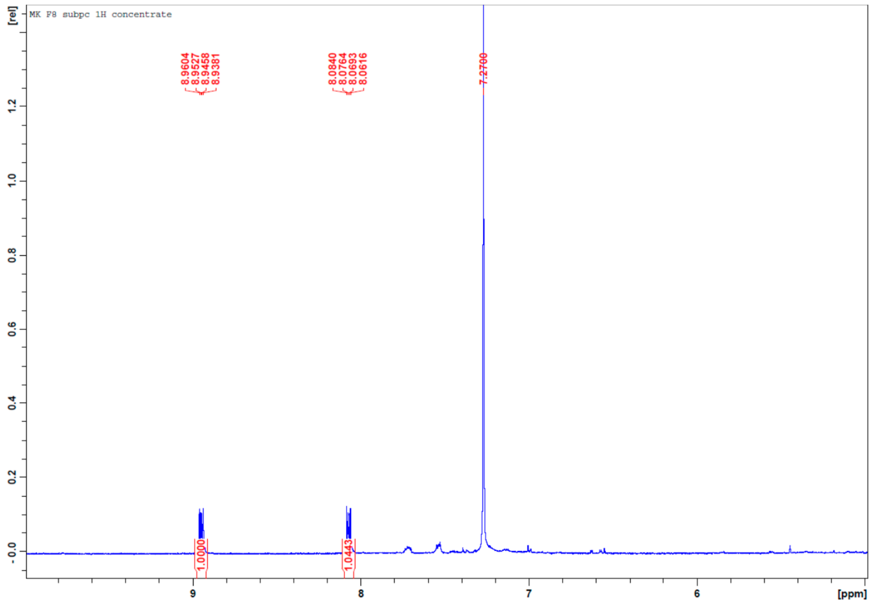

Figure S8 – ^{1}H NMR spectrum for SPc(A_2B) showing two doublets of doublets (ortho and meta coupling).
